# Enhancing docetaxel efficacy and reducing toxicity using biodegradable periodic mesoporous organosilica nanoparticles

**DOI:** 10.1016/j.heliyon.2024.e40131

**Published:** 2024-11-05

**Authors:** Ha Nguyen Van, Linh Ho Thuy Nguyen, Ngoc Xuan Dat Mai, Anh Ha Nhat, Trinh Le Thi Thu, Anh Nguyen Thi Bao, Ha Nguyen Thanh, Minh Tri Le, Tan Le Hoang Doan

**Affiliations:** a University of Health Science (UHS), VNU-HCM, Ho Chi Minh City, Viet Nam; b Faculty of Pharmacy, University of Medicine and Pharmacy at Ho Chi Minh City, Ho Chi Minh City, Viet Nam; c Center for Innovative Materials and Architectures (INOMAR), Ho Chi Minh City, Viet Nam; d Vietnam National University, Ho Chi Minh City, Viet Nam; e Institute of Drug Quality Control Ho Chi Minh City (IDQC HCMC), Viet Nam

**Keywords:** Docetaxel, Nanomaterial, Cancer, Drug delivery, BPMO, MSN

## Abstract

Taxanes, such as docetaxel (DTX), are pivotal in cancer therapy, showcasing remarkable efficacy against various cancers, like breast, lung, and ovarian malignancies. However, DTX's efficacy is hindered by poor target specificity and significant adverse effects. Formulations containing DTX often include polysorbate 80 and ethanol, exacerbating reactions like hypersensitivity and neurological disorders. Nanotechnology offers a promising avenue to address these challenges, aiming to enhance DTX's targeted delivery and solubility. Mesoporous silica nanoparticles (MSN), notably biodegradable periodic organosilane (BPMO), have emerged as promising carriers due to their stability, biocompatibility, and drug-loading capacity. BPMO's intracellular biodegradability reduces the risk of toxic accumulation. Compared to conventional MSN, BPMO particles exhibit superior characteristics, including size, surface area, and DTX loading ability. Moreover, cell line studies suggest BPMO's potential to mitigate DTX-associated adverse effects. These findings highlight BPMO nanoparticles' potential in improving DTX delivery, solubility, and reducing adverse effects, underscoring their significance in cancer therapy.

## Highlights

• Enhanced DTX loading capacity and improved solubility with BPMO nanoparticles.
• Significant reduction in adverse effects associated with DTX, as demonstrated in cell line studies.
• Optimized BPMO particle size and surface area enhance their effectiveness as drug carriers.

## Graphical abstract

**Figure:**
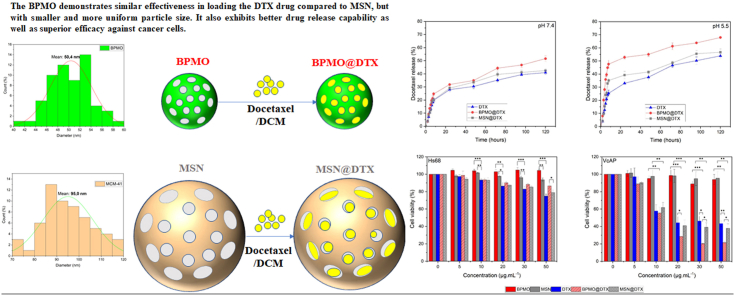

## 1 Introduction

Docetaxel (DTX), a semi-synthetic anticancer agent belonging to the taxane family, is extracted from the European yew tree (*Taxus baccata*) and has applications in the treatment of various cancers, including prostate, ovarian, and breast cancers [1]. It functions by inhibiting microtubules, thereby disrupting cancer cell division [2]. However, DTX has several limitations, including poor target specificity, high systemic toxicity, and low water solubility, which reduce its treatment efficacy [3,4].

To overcome these shortcomings, nano-technology, including lipid-based nanocarriers, inorganic nanoparticles, and polymer nanoparticles, has been recently applied to DTX and has been shown to significantly improve DTX's properties and therapeutic effects [5,6]. Mesoporous silica nanoparticles (MSN), which have several advantages, including high stability, good biocompatibility, non-toxicity, high porosity, nanoscale size, high uniformity, and high drug-loading capacity, have recently emerged. Because of their surface functionalization capability, MSNs can be used as carriers for targeted drug delivery and controlled release [7,8,9].

Among the non-biodegradable nanoparticles, MSN is the most extensively researched silica particle because of its numerous advantages, including a high framework order, narrow size distribution, cylindrical pore structure, large specific surface area, a facile synthesis process, and flexibility and tunability to generate nanoparticles with different sizes, structures, morphologies, and surface properties [10]. Only the study by Eva Rivero-Buceta et al. (2019) [11] has been found to apply MSN to DTX, where a covalent conjugate of DTX and prostate-specific membrane antigen (PSMA) targeting molecule was developed and attached to MSN, generating MCM-41. The results showed that nanoparticles containing PSMA-conjugated DTX increased cancer cell cytotoxicity by twofold [11]. However, this study used PSMA-conjugated DTX and not pristine DTX.

When considering clinical treatment, the carrier material's biodegradability needs to be thoroughly evaluated because only <6 nm particles can be excreted through the kidneys, while particles >8 nm are not excreted, leading to higher toxicity [12,13]. Since 2021, Ngoc Xuan Dat Mai and colleagues have developed a new type of biodegradable periodic organosilane (BPMO) particles that combine the advantages of hybrid inorganic–organic materials with the unique characteristics of cyclic porous materials, including biodegradability. In BPMO, the organic molecules incorporated into the porous silica framework respond excellently to various intracellular conditions, including low pH, presence of enzymes, and oxidative processes, thereby leading to BPMO particle degradation and elimination [14,15]. BPMO has been used to load and release various substances, including curcumin [14] and cordycepin [16], and it has demonstrated high drug-loading capacity, good biodegradability, and enhanced active ingredient efficacy. Regarding paclitaxel, a taxane that is structurally similar to DTX, Mai and colleagues assessed BPMO biodegradability, loading capability, and *in vitro* cytotoxicity. Their analyses showed that BPMO particles were rapidly degraded in the test environment and that they had an extremely high loading capacity for hydrophobic paclitaxel molecules, which improved paclitaxel's anticancer activity [17]. Thus, BPMO has potential applications for DTX loading, biodegradation, and enhanced efficacy. However, since no studies have been conducted on DTX and BPMO, a comprehensive evaluation is needed.

In this study, the DTX-carrying abilities of MSN and BPMO in cancer treatment were evaluated and compared. Scanning electron microscopy (SEM), nitrogen adsorption-desorption isotherm analysis, Fourier-transform infrared spectroscopy (FTIR), and thermogravimetric analysis were used for nanoparticle characterization. DTX loading capacity onto the two types of nanoparticles was examined at various concentrations, in different loading solvents, and at different loading times. DTX's release over 2–120 h was evaluated in a simulated release environment at pH 7.4 and 5.5. The MTT assay was used to assess the anticancer properties of MSN@DTX and BPMO@DTX in normal Hs68 cells, the prostate cancer cell line, VcaP, the human lung adenocarcinoma cell line, A549 and the breast cancer cell line, MCF-7, to demonstrate DTX's improved anticancer activity. This study is novel because of the absence of studies comparing MSN's and BPMO's drug loading and release characteristics and cytotoxicity to demonstrate the advantages of biodegradable BPMO as a DTX carrier in cancer treatment. This research may offer new insights into the development of a novel biodegradable material for DTX loading and targeted distribution, thereby reducing toxicity.

## 2 Experimental

### 2.1 Materials

The following analytical grade reagents were used as received from commercial suppliers without further purification: hexadecyltrimethylammonium bromide (CTAB, 99+%, Acros Organic), tetraethyl orthosilicate (98 %, Acros Organic), 1M sodium hydroxide (Fisher), ethanol (99.8 %, Fisher), acetonitrile for HPLC (99.9 %, Merck), ammonium chloride (99.5 %, Fisher), phosphate-buffered saline (PBS, pH 7.4, Sigma–Aldrich), citrate–phosphate-buffer (CPB, pH 5.5, Sigma–Aldrich), tween-80 (Xilong), 1,2-bis(triethoxysilyl)ethane (Sigma–Aldrich), bis[3-(triethoxysilyl)propyl] tetrasulfide (Sigma–Aldrich), concentrated hydrochloric acid (Sigma–Aldrich), dichloromethane (DCM), dimethyl sulfoxide (DMSO) (Sigma–Aldrich), and DTX (Sigma–Aldrich). All solutions were prepared using Milli-Q water.

### 2.2 Nanoparticle synthesis

#### 2.2.1 MSN synthesis

A mixture of CTAB (250 mg), Milli-Q water (120 mL), and 1M NaOH (1,752 μL) was prepared in a 250 mL round-bottom flask. The solution was stirred at 80 °C, at 1,500 revolutions per minute (rpm). After temperature stabilization, 1,250 μL of tetraethyl orthosilicate was slowly added into the flask in a dropwise manner. After a 2-h reaction, stirring was continued until the mixture cooled to room temperature. Next, the mixture was centrifuged for 30 min at 25 °C (15,000 rpm), followed by precipitate collection. The precipitate was then washed twice with absolute ethanol. For complete CTAB removal, the precipitate was refluxed overnight with a solution of 1 mL concentrated hydrochloric acid in absolute ethanol (sufficient to make 50 mL of solvent) and stirred at 500 rpm at 85 °C. The product was then cooled and centrifuged for 30 min at 25 °C (15,000 rpm), followed by precipitate collection. The precipitate was then washed thrice with absolute ethanol and dried at 80 °C for 24 h [18].

#### 2.2.2 BPMO synthesis

BPMO particles were synthesized as we previously described [16]. A mixture of CTAB (250 mg), Milli-Q water (120 mL), and 1M NaOH (800 μL) was prepared in a 250 mL round-bottom flask and stirred at 80 °C, at 1,500 rpm. After temperature stabilization, 300 μL of 1,2-bis(triethoxysilyl)ethane was slowly added in a dropwise manner, followed by the addition of 100 μL of bis[3-(triethoxysilyl)propyl]tetrasulfide. After 2 h of reaction, the mixture was cooled to room temperature while stirring, followed by centrifugation (15,000 rpm) for 30 min at 25 °C and precipitate collection. The precipitate was then washed twice with absolute ethanol. For complete CTAB removal, the precipitate was refluxed overnight in a solution of ammonium chloride (300 mg) in distilled water (2 mL), and supplemented with absolute ethanol to a final volume of 50 mL while stirring (500 rpm) at 85 °C. After cooling, the product was centrifuged (15,000 rpm) for 30 min at 25 °C. The precipitate was then collected, washed thrice with absolute ethanol, and dried at 80 °C for 24 h.

### 2.3 Characterization of nanoparticle

SEM was conducted using a Hitachi S4800 microscope. The zeta potential and dynamic light scattering (DLS) were conducted using Zetasizer Nano ZS (Malvern, UK). Nitrogen adsorption–desorption isotherms were measured on a Quantachrome Autosorb iQ2 physisorption analyzer, with a temperature condition of 77 K being maintained using ultra-pure N_2_ and He (99.999 % purity). The samples’ FTIR spectra in the 4000–400 cm^−1^ range were measured using a Bruker Vertex 70 FTIR spectrometer. Thermogravimetric analysis was done using A TA Instruments Q-500 thermal gravimetric analyzer, with a continuous nitrogen flow being maintained from room temperature to 800 °C at a heating rate of 5 °C/min.

### 2.4 Drug loading and release analyses

#### 2.4.1 Solvent influence on loading capacity

DTX was loaded onto nanoparticles using the adsorption equilibrium method [19]. Briefly, 1 mg of the nanomaterial was added to 1 mL of DTX (1 mg/mL) in the respective solvents. The resulting mixture was then sonicated for uniform dispersion and stirred at 600 rpm for 24 h at room temperature. The mixture was centrifuged for 30 min at 25 °C (15,000 rpm). The supernatant was then collected for further analysis, while the DTX-loaded nanoparticles were air-dried. The supernatant containing DTX in ethanol and DMSO was filtered through a 0.45-μm membrane filter and the residual DTX content in the supernatant was determined using high-performance liquid chromatography (HPLC) with a photodiode array (PDA) detector. The supernatant containing DTX in DCM was dried at room temperature and the dried DTX residue was re-dissolved in a mixture of acetonitrile and water (50:50). The resulting supernatant was then treated in the same way as those in ethanol and DMSO solvents.

The loading capacity of the nanomaterial was calculated based on the residual DTX content in the supernatant. HPLC analyses were done under the following parameters on a SHIMADZU Prominence-I LC-2030C 3D HPLC system equipped with a PDA detector (190–800 nm) and an autosampler:

• A 25-cm x 4.6-mm column with a 5-μm particle size.
• An acetonitrile–water (60:40, v/v) mobile phase.
• Wavelength: 230 nm.
• Injection volume: 20 μL.
• Temperature: 30 °C.
• Flow rate: 1.2 mL/min.

Drug-loading performance and capacity were calculated using the following formulas:

(1) $$•\;\text{Drug}\;\text{loading}\;\text{performance}\;\left(\%\right)=\frac{m_{0}-m_{r}}{m_{0}}\times 100$$

(2) $$•\;\text{Drug}\;\text{loading}\;\text{capacity}\;\left(\text{mg}/g\right)=\frac{m_{0}-m_{r}}{m_{\text{NPs}}}$$

where m_0_ (mg) is the initial DTX mass loaded onto nanoparticles, m_r_ (mg) is the DTX mass remaining in the supernatant, and m_NPs_ (g) is the nanoparticle mass used for loading.

#### 2.4.2 The effect of the initial DTX concentration on the nanomaterials’ loading capacities

This analysis was similar to the above-described experiment on the effect of solvents, but DTX was used in the solvent at concentrations of 0.25, 0.5, 0.75, 1.0, 1.25, and 1.5 mg/mL to determine the optimal loading concentration and compare the nanoparticle types.

#### 2.4.3 The influence of the drug-to-nanomaterial mass ratio on the nanomaterials’ loading capacities

After determining the optimal initial DTX concentration, this analysis was similar to the above-described solvent influence experiment, but DTX and nanomaterials were used at ratios of 1:1, 1:2, 1:3, 1:4, and 1:5 to determine the ratio with the highest loading efficiency and compare the two nanoparticle types.

#### 2.4.4 The effect of time on the nanomaterials’ drug loading capacity

After determining the solvent type, initial DTX concentration, and the optimal drug-to-nanomaterial ratio, an experiment similar to the above-described solvent influence analysis was conducted. However, drug loading capacity was evaluated at 2, 4, 8, 12, 24, 36, and 48 h to determine the nanomaterials’ adsorption equilibrium time and compare the two nanoparticle types.

#### 2.4.5 *In vitro* drug release analysis

The *in vitro* DTX release capability was assessed using the dialysis bag diffusion method [20]. BPMO@DTX, MSN@DTX, and pure DTX (same amount as the amount loaded onto the nanomaterials) samples were dispersed via ultrasonication in 10 mL of a release medium containing PBS + 0.1 % (v/v) tween 80 (pH 7.4) and then placed into the diffusion cells and sealed tightly at both ends. Next, the diffusion cells were immersed in a release medium (60 mL) in stoppered Erlenmeyer flasks with continuous shaking (100 rpm) at 37 °C. Next, 1 mL of the release medium was withdrawn after 2, 3, 4, 5, 6, 7, 8, 24, 48, 72, 96, and 120 h and replaced with 1 mL of fresh medium. At each time point, the amount of DTX released into the solution was quantified using HPLC. The DTX concentration in the release medium was calculated using a linear regression equation. The drug release efficiency was determined using formula (3).

(3) $$H\%\;\left(t\right)=\frac{m_{r}\;\left(t\right)}{m_{l}}\times 100\;\%$$

where H%(t) is the drug release efficiency at time t, m_r_(t) is the amount of drug released at time t (mg), and m_l_ is the DTX mass loaded onto the nanoparticles (mg).

For CPB +0.1 % (v/v) tween 80 medium (pH 5.5), the process is carried out similarly.

### 2.5 Cytotoxicity

The anticancer efficacy and cytotoxic effects of DTX, MSN@DTX, and BPMO@DTX were evaluated using the MTT (3-[4,5-dimethylthiazol-2-yl]-2,5-diphenyl tetrazolium bromide) assay. Unloaded BPMO and MSN samples were also tested for comparison. This assay relies on the reduction of MTT (yellow) to formazan (purple) by mitochondria in viable cells, with the colorimetric comparison indicating the number of surviving cells [21]. These analyses used the normal human fibroblast cell line Hs68 (source ATCC, supplier Sigma-Aldrich), the prostate cancer cell line VcAP (source ATCC, supplier Sigma-Aldrich), the human lung adenocarcinoma cell line A549 (source ATCC, supplier Sigma-Aldrich) and the breast cancer cell line MCF-7 (source ATCC, supplier Sigma-Aldrich). The cells were seeded on 96-well plates at a density of 5 × 10^4^ cells/mL in Dulbecco's Modified Eagle's Medium (Gibco) supplemented with 10 % fetal bovine serum (Gibco), penicillin (100 IU/mL), and streptomycin (100 μg/mL), and then cultured at 37 °C (5 % CO_2_) for 24 h. Next, the cells were treated with the test samples at different concentrations, as well as the control. A solution containing phosphate buffer and 0.1 % (v/v) tween 80 (pH 5.5) was chosen as the sample preparation medium. Dilute the test samples to the experimental concentration with buffer solution.

After 72 h, 10 μL of MTT (5 mg/mL) were added to each well. The cells were then incubated for 4 h at 37 °C (5 % CO_2_). The medium in the wells was then removed, followed by DMSO addition to dissolve the formazan crystals. Absorbance was then measured at 570 nm on a microplate reader (Thermo Scientific™, Varioskan™, USA). The percentage of dead cells was determined using formula (4).

(4) I(%)=100−100×A/C

where I (%) is the percentage of dead cells, A is the optical density of the test sample, and C is the optical value of the control sample. The invert value of I is the percentage of live cells.

The IC50 value of each type of nanoparticle was determined based on the concentration–cell viability (%) curve. Based on this IC50 value, the Selectivity Index (SI) was calculated to assess the selective cytotoxicity of nanoparticles toward cancer cells in comparison to normal cells. The SI value was calculated using the following formula (5):

(5) SI = (IC50 of normal cells)/(IC50 of cancer cells)

Here, “normal cells” refer specifically to the Hs68 cell line, while “cancer cells” include the VcAP, A549, and MCF-7 cell lines [22].

## 3 Results and discussion

### 3.1 Nanoparticle characterization

**Fig. 1:**
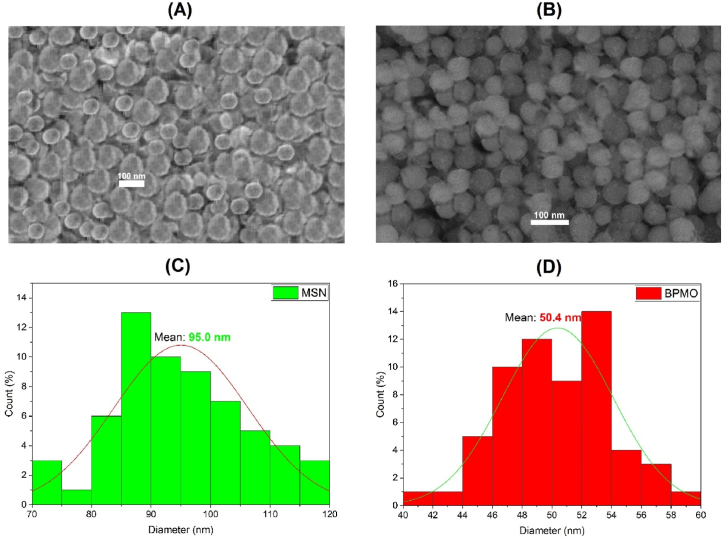
SEM images of MSN (A) and BPMO (B) nanoparticle. Particle size distribution of MSN (C) and BPMO (D) was determined using ImageJ.

We synthesized two types of tetrasulfide-based porous organosilica nanoparticles using the sol-gel method as previously described [14]. SEM analysis of MSN particles (Fig. 1A) revealed uniform spherical nanoparticles with an average particle size of about 100 nm. Additionally, as shown in Fig. 1C, the nanoparticles were distributed within a narrow bell-shaped region, with an average size of about 95 nm. These sizes are consistent with our previous findings [23]. SEM analysis of BPMO (Fig. 1B) revealed uniform spherical nanoparticles with an average size of about 50 nm that seemed more homogeneous than MSN particles. This observation is further supported by the observation that BPMO nanoparticles are distributed in a narrower bell-shaped region (ranging from 40 to 60 nm as opposed to 70–120 nm for MSN), with an average size of about 50.4 nm (Fig. 1D). It can be seen in Fig. 1 that the BPMO particle size is only half the size of MSN particles and they are more evenly distributed, which enhances stability when loading active substances. Additionally, as suggested by Wenqi Yu et al. (2020) [24], smaller nanoparticles have better permeability and can more effectively penetrate tumors, thereby improving treatment efficacy.

**Fig. 2:**
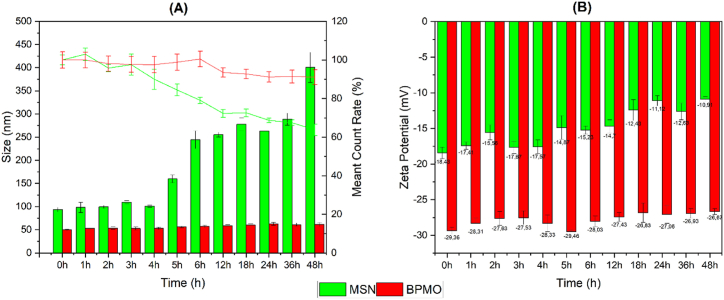
The average particle size and count rates of the MSN (A) and BPMO (B); the Zeta potentials of the MSN (C) and BPMO (D).

To assess and compare the stability of MSN and BPMO nanoparticles, we dispersed both types of nanoparticles in deionized water and subjected the samples to 60 min of ultrasonication (Elma P30H, 37 kHz, 350 W). Dynamic Light Scattering (DLS) and zeta potential measurements were then taken at multiple time points over a 48-h period, with the samples maintained at room temperature (25 °C) between measurements. The results showed that MSN nanoparticles (Fig. 2A) had an initial particle size similar to that observed in Scanning Electron Microscopy (SEM) images (approximately 93.4 nm) and remained stable for the first 4 h. However, after 5 h, the particle size gradually increased, reaching a peak of approximately 400.5 nm by the 48-h mark, indicating a significant loss of stability beyond 5 h. Additionally, the count rate dropped substantially to below 70 % by the end of the 48-h period. In contrast, BPMO nanoparticles exhibited consistent stability, with an initial particle size of around 49.8 nm that only slightly increased to 61.0 nm by the 48-h time point. The count rate for BPMO remained stable throughout, with no notable fluctuations, consistently staying above 90 %.

To further evaluate stability, we measured the zeta potential of both MSN and BPMO nanoparticles (Fig. 2B). The results showed that BPMO nanoparticles maintained a consistently stable and strongly negative zeta potential, starting at −29.36 mV and slightly decreasing to −26.67 mV after 48 h. These values are consistent with the findings of Mai et al. [23]. In contrast, MSN nanoparticles exhibited a higher but less stable zeta potential, beginning at −18.43 mV and decreasing to −10.91 mV over the same period. These results suggest that BPMO nanoparticles possess greater stability compared to MSN nanoparticles.

**Fig. 3:**
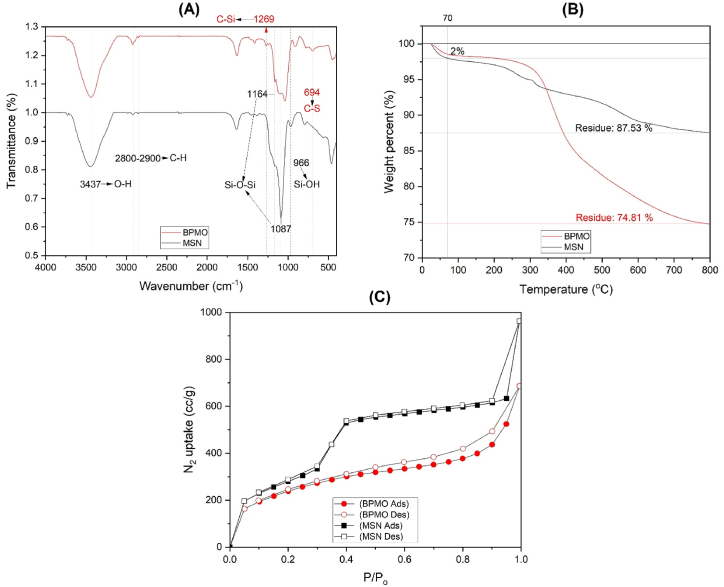
(A) The FTIR spectra, (B) thermogravimetric analysis, and (C) isothermal adsorption–desorption of MSN and BPMO.

FTIR analysis was used to identify nanoparticle functional groups. As shown in Fig. 3A, MSN (black line) has a broad peak at 3437 cm^−1^, which corresponds to the stretching vibration of surface O–H bonds because of water adsorption on the nanomaterial's surface or silanol groups [25]. Absorption peaks at 1164 cm^−1^ and 1087 cm^−1^, which correspond to the stretching vibration of Si–O–Si bonds in siloxane groups, indicate successful condensation [26]. Additionally, the peak at 966 cm^−1^ is characteristic of the Si–OH bond's (silanol) stretching vibration [27]. The absorption peak at around 2800–2900 cm⁻^1^, which corresponds to C–H stretching vibrations, indicates residual CTAB presence [28]. Some BPMO peaks, such as the peaks at 3437 cm^−1^ (which corresponds to O–H groups) [25], 1036 cm^−1^, and 1160 cm^−1^ (which correspond to Si–O–Si groups) [26], and the absorption peak at around 2800–2900 cm^−1^ (residual CTAB), are similar to the MSN spectrum [28], indicating that BPMO and MSN are structurally similar. However, BPMO has structural differences observed at 694 cm⁻^1^, which corresponds to the C–S bond [29], and the absorption peak at 1269 cm⁻^1^, which is characteristic of C–Si stretching vibration [30]. These differences result from adding the bis[3-(triethoxysilyl)propyl] tetrasulfide framework stabilizer during BPMO synthesis, which introduces C–S and C–Si structures. The FTIR spectra indicate that the typical functional groups of both nanoparticles were successfully condensed. All the obtained peaks were consistent with previous findings [23,17].

Thermogravimetric analysis was used to further characterize the composition of the nanoparticles. As shown in Fig. 3B, both types of nanoparticles lose a small amount of weight (approximately 2 %) at up to 70 °C, which corresponds to the adsorption of solvents and water within the porous structures. At around 250–800 °C, a significant amount of material decomposes. In both nanoparticles, this decomposition corresponds to organic components, ethane- and tetrasulfide-moiety structures within the silica framework [23] and confirms successful bonding structure synthesis.

The obtained nitrogen adsorption-desorption isotherms were also used to evaluate nanoparticle porosity. The isotherms revealed that both nanomaterials exhibit type IV mesoporous structures (Fig. 3C) [23]. Therefore, MSN's Brunauer–Emmett–Teller surface area was calculated to be 1,394.738 m^2^/g, its pore diameter was approximately 2.909 nm, and its pore volume was 1.577 cm³/g based on the Barrett–Joyner–Halenda theory. Similarly, BPMO had a Brunauer–Emmett–Teller surface area of 799.515 m^2^/g, a pore diameter of approximately 1.810 nm, and a pore volume of 1.007 cm³/g based on the Barrett–Joyner–Halenda theory. These results confirm successful nanoparticle synthesis and are consistent with the findings of previous studies [31].

### 3.2 DTX loading capacity onto nanoparticles

#### 3.2.1 The influence of solvents on loading capacity

**Fig. 4:**
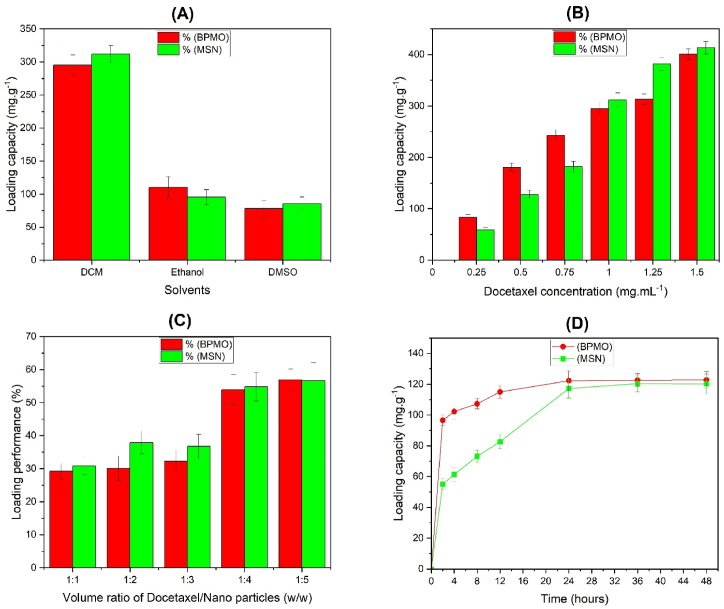
Loading capacity onto BPMO and MSN. (A) Loading capacity based on solvent. (B) Loading capacity based on DTX concentration. (C) Loading performance based on the DTX-to-nanoparticle volume ratio. (D) Loading capacity based on time.

DTX was loaded onto the nanoparticles using the adsorption equilibrium method. DTX's loading capacity onto MSN and BPMO nanoparticles was investigated in the following solvents: DCM, ethanol, and DMSO, at a ratio of 1 mg of nanoparticles to 1 mg/mL of DTX in the solvent. This analysis revealed that for MSN, the highest amount of DTX (loading capacity: 311.88 mg/g) was loaded with DCM as solvent and the loading capacity decreased to 95.25 and 85.13 mg/g with ethanol and DMSO, respectively (Fig. 4A). It can be observed from Fig. 4A that the drug loading capacity increases with decreasing polarity index (the polarity index of DMSO > ethanol > DCM), probably because the less polar solvent does not compete with the highly hydrophobic DTX molecules for adsorption onto the MSN surface [20]. Thus, different solvents can be used to control drug loading capacity onto MSN, with DCM yielding the highest loading capacity. This is consistent with the findings by Yongju He et al. (2017) about loading paclitaxel (along with DTX) onto MSN [20]. Similarly, for BPMO, the highest loading capacity (295.24 mg/g) was achieved with DCM, followed by ethanol at 110.20 mg/g and DMSO at 78.09 mg/g. Therefore, for both nanoparticles, DCM use as solvent had the highest drug loading efficiency and DCM's DTX loading capacity did not differ significantly between MSN and BPMO (311.88 and 295.24 mg/g, respectively), although MSN had a larger surface area, pore diameter, and pore volume (about 1.5 times larger). This is attributable to MSN's outer surface, which has numerous hydrophilic -OH groups, as well as uneven particle sizes, which limits DTX's adsorption capacity when compared with BPMO particles that lack -OH groups on the outer surface and are more evenly distributed [10,14]. This indicates that although BPMO's average particle size (approximately 50 nm) is smaller than MSN's (100 nm) DTX's loading capacity onto BPMO is comparable to that of MSN. Based on these data, we chose DCM as the solvent for further investigation.

#### 3.2.2 The influence of the initial DTX concentration on the loading capacity

Next, we used DTX at concentrations (in the solvent) of 0.25, 0.5, 0.75, 1.0, 1.25, and 1.5 mg/mL while keeping the nanoparticle mass constant (1 mg). As shown in Fig. 4B, MSN's loading capacity increased with increasing DTX concentration and the loading capacity was highest (413.29 mg/g) at a DTX concentration of 1.5 mg/mL. Similarly, BPMO's loading capacity was highest (400.67 mg/g) at a DTX concentration of 1.5 mg/mL. At low concentrations of DTX (<1 mg/mL), BPMO's DTX loading capacity was better than MSN's and this trend was reversed at concentrations of >1 mg/mL. This is probably because when compared with MSN, BPMO has a stronger affinity for DTX and reaches saturation earlier because of its higher oil affinity [32], which makes it more suitable for the adsorption of DTX, a strongly lipophilic substance [33]. At a DTX concentration of 1.0 mg/mL, the loading capacities of MSN and BPMO were 311.89 and 295.24 mg/g, respectively. This difference, which is not significant, still indicates the significant DTX loading potential of BPMO when compared with MSN.

#### 3.2.3 The influence of the drug-to-nanomaterial mass ratio on loading capacity

Next, a DTX concentration of 1.0 mg/mL was chosen to investigate loading capacity at drug-to-nanomaterial mass ratios of 1:1, 1:2, 1:3, 1:4, and 1:5. Fig. 4C shows that MSN's loading capacity increased with increasing drug-to-nanomaterial mass ratio and was saturated at the 1:4 ratio, peaking at the 1:5 ratio, with a loading efficiency of 56.68 %. Similarly, BPMO's loading capacity reached saturation at the 1:4 ratio and peaked at the 1:5 ratio, with a loading efficiency of 56.95 %. These data indicate that for MSN and BPMO nanoparticles, the loading efficiency is highest at the drug-to-nanomaterial mass ratio of 1:5 and is not significantly different between MSN and BPMO. These results further confirm BPMO's DTX loading potential when compared with MSN.

#### 3.2.4 The influence of time on loading capacity

Next, the drug-to-nanomaterial mass ratio of 1:5 was chosen to investigate loading capacity at 2, 3, 4, 5, 6, 7, 8, 24, and 48 h. Fig. 4D shows that the DTX adsorption process onto MSN and BPMO reached equilibrium after 36 h (loading capacity: 120.33 mg/g) and 24 h (loading capacity: 122.25 mg/g), respectively. Because BPMO has a large surface area and a pore size conducive to the diffusion process of DTX to the adsorption centers of the nanomaterial, DTX adsorption capacity increased during the initial period. Additionally, during the initial period, there were more empty adsorption centers in the pores and DTX concentration on BPMO was much lower than in the solution. The adsorption process tended to reach equilibrium at 24 h, as the number of adsorption centers and empty pores decreased significantly. Considering the time before reaching equilibrium, BPMO exhibited a higher loading capacity than MSN at earlier time points, including at 2 h (loading capacity: 96.51 % vs 55.12 % for BPMO vs MSN) and 4 h (loading capacity: 102.23 % vs 61.31 % for BPMO vs MSN). Because BPMO reaches saturation earlier (at 24 h) than MSN (at 36 h), and it has a higher loading capacity at early time points, DTX loading onto BPMO is more favorable because it reduces agitation time.

Although these results indicate that considering the loading capacity aspect, DTX loading onto BPMO is comparable to MSN's, it has the advantages of smaller particle size and earlier saturation, which can minimize solvent wastage and save time.

To confirm that there were no changes in particle morphology and size after loading DTX onto BPMO and MSN particles, SEM imaging was conducted on BPMO@DTX and MSN@DTX (Fig. S1). The results indicated that MSN@DTX (Fig. S1A) and BPMO@DTX (Fig. S1B) maintained their original spherical morphology and particle size, consistent with the initial MSN and BPMO nanoparticles, respectively.

### 3.3 DTX *in vitro* release profile

MSN@DTX, BPMO@DTX, and free DTX particles were used to evaluate DTX release into PBS (pH 7.4) and CPB (pH 5.5), both containing 0.1 % (v/v) Tween 80 to enhance DTX's solubility [34]. Based on the DTX loading capacity of 122.25 mg/g for BPMO and 120.33 mg/g for MSN, the DTX weight was calculated to correspond to 1 mg of free DTX. The weight of MSN@DTX was 8.31 mg and the weight of BPMO@DTX was 8.18 mg.

**Fig. 5:**
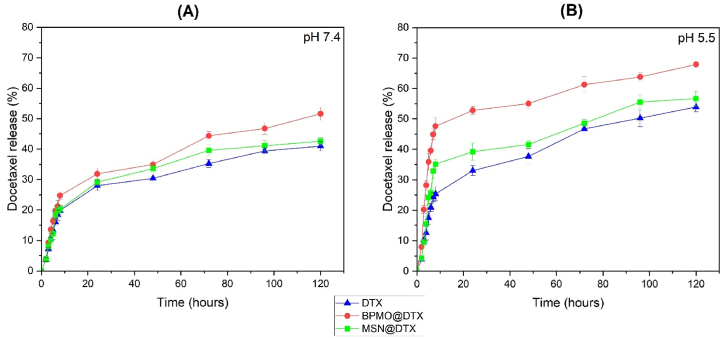
(A). DTX release (%) at pH 7.4 (A) and pH 5.5 (B).

Fig. 5A shows that at pH 7.4, drug release was not significantly different between MSN@DTX and free DTX. The release process increased gradually and maximized at 120 h. However, the BPMO@DTX sample exhibited superior release kinetics, and when compared with MSN@DTX and free DTX, its release was higher as early as after 3 h, and remained consistently higher at all time points, peaking at 51.6 % at 120 h when compared with 42.55 % and 40.95 % for MSN@DTX and free DTX, respectively. This indicates that at pH 7.4, BPMO improves the solubility of free DTX and has a higher DTX-solubilizing capacity than MSN.

Fig. 5B shows that as at pH 7.4, the MSN@DTX's release profile was similar to that of free DTX at pH 5.5, steadily increasing and peaking at 120 h. When compared with free DTX, MSN@DTX exhibits a slightly higher release starting at 4 h, although the difference in release is not significant. However, when compared with free DTX and MSN@DTX, BPMO's release profile was higher from the start of the evaluation and remained consistently high, peaking at 120 h at 67.91 %, when compared with 56.69 % and 53.91 % for MSN@DTX and free DTX, respectively.

A comparison of both release profiles at pH 7.4 and 5.5 revealed that MSN@DTX's release was not significantly improved when compared with free DTX. However, BPMO@DTX's release capability was significantly higher when compared with free DTX. The higher DTX release from BPMO at pH 5.5 than at pH 7.4 is probably because the initial BPMO synthetic design is prone to degradation in low pH environments [35]. These observations indicate that when compared with MSN, BPMO has a significantly greater capacity to improve DTX solubility in pH 7.4 and pH 5.5 environments.

### 3.4 Cytotoxicity

Next, we used the MTT assay to examine the cytotoxicity of various MSN@DTX, BPMO@DTX, and free DTX concentrations on the normal fibroblast cell line, Hs68, to determine if they are harmful to normal cells. Moreover, the prostate cancer cell line, VcAP, was used to evaluate the cancer cell cytotoxicity of MSN@DTX, BPMO@DTX, and free DTX. The impact of free MSN and BPMO on the cells was also evaluated.

**Fig. 6:**
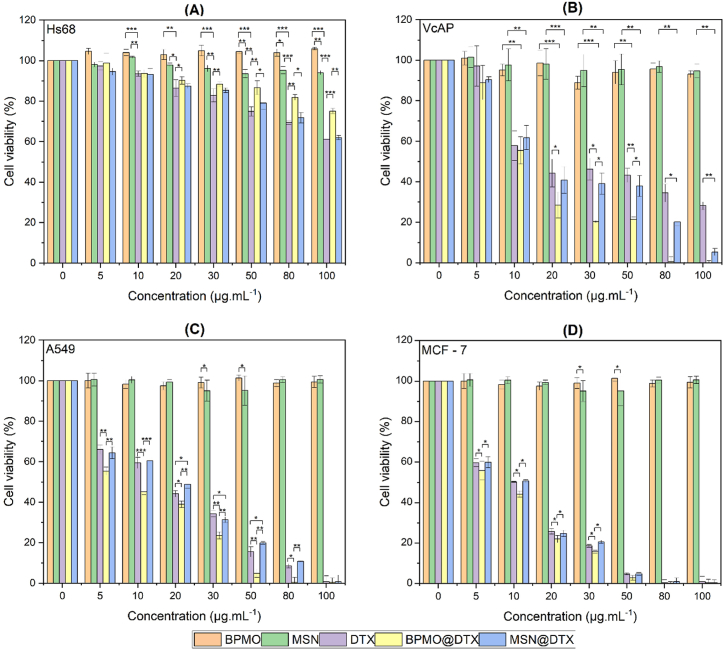
The viability (%) of (A) Hs68 fibroblast cells; (B) VcaP prostate cancer cells; (C) A549 human lung adenocarcinoma cells and (D) MCF-7 breast cancer cells treated with BPMO, MSN, DTX, BPMO@DTX, and MSN@DTX was evaluated using the MTT assay. N = 3. ∗, ∗∗, and ∗∗∗ indicate p < 0.05, <0.01, and <0.001, respectively (Student t-test).

Fig. 6A shows that free DTX, nanoparticles, and DTX-loaded nanoparticles have mild toxicity on Hs68 cells, with the percentage of viable cells decreasing gradually with increasing concentrations, although not significantly. Notably, in Hs68 cells, when compared with MSN@DTX, BPMO@DTX was associated with consistently higher cell viability at all concentrations, and free BPMO hardly exhibited any toxicity to Hs68 cells. When comparing the cell viability between BPMO@DTX and free DTX, at concentrations of 20 μg/mL and above, the difference becomes significant and statistically meaningful (with p < 0.05 at 20 μg/mL and p < 0.01 at 30 μg/mL and higher), indicating that BPMO@DTX reduces toxicity on normal Hs68 cells compared to free DTX. This can be explained by the fact that free DTX exists in solution form with a release rate considered to be absolute (100 %), unlike DTX in BPMO@DTX, which exhibits a slower release rate due to the obstruction by BPMO nanoparticles during the release process (Fig. 5). A comparison between BPMO@DTX and MSN@DTX reveals that the difference in cell viability starts to emerge from concentrations of 30 μg/mL and above (with p < 0.01), indicating that BPMO@DTX reduces toxicity on normal Hs68 cells compared to MSN@DTX. These findings suggest that, in normal Hs68 cells, BPMO@DTX significantly reduces toxicity compared to both MSN@DTX and free DTX.

Fig. 6B shows that the viability of VcAP prostate cancer cells treated with BPMO@DTX decreased steadily with increasing BPMO@DTX concentration, with the most significant viability decrease starting at 20 μg/mL, indicating increasing toxicity against VcaP cells. When compared with free DTX, at low concentration (5 μg/mL of DTX), BPMO@DTX was associated with lower cell viability and this difference became more pronounced at higher concentrations. When compared with BPMO@DTX-treated VcaP cells, cell viability at concentrations from 10 μg/mL was consistently higher in MSN@DTX-treated cells, indicating that VcaP inhibition by MSN@DTX was lower. This is probably because of the higher DTX release by BPMO@DTX when compared with MSN@DTX in the experimental conditions. However, cell viability was not significantly different upon treatment with MSN@DTX when compared with DTX, suggesting that MSN@DTX did not have improved anticancer efficacy. This is probably because MSN@DTX does not significantly enhance DTX solubility in the test conditions. Both free BPMO and MSN nanoparticles did not exhibit toxicity against Hs68 cells. These results offer compelling evidence that BPMO@DTX has superior anticancer activity than free DTX and MSN@DTX, and suggest that BPMO has promising potential applications for the advancement of drug delivery and cancer therapy.

In the cytotoxicity test on the A549 lung cancer cell line, Fig. 6C demonstrates a significant difference in the cytotoxicity of BPMO@DTX compared to free DTX and MSN@DTX. Specifically, at a concentration as low as 5 μg/mL, the cytotoxicity of BPMO@DTX increased significantly compared to free DTX (with p < 0.01), and this difference became more pronounced at higher concentrations. Unlike BPMO@DTX, MSN@DTX did not show a statistically significant difference from free DTX at lower concentrations, with an increase in cytotoxicity only observed at concentrations of 20 μg/mL and above. These results indicate that BPMO@DTX enhances the anticancer efficacy in A549 cells compared to both free DTX and MSN@DTX.

The same results were also observed in the MCF-7 breast cancer cell line (Fig. 6D); however, MSN@DTX showed no statistically significant difference compared to free DTX, despite both exhibiting cytotoxic effects starting from a concentration of 5 μg/mL. Conversely, for BPMO@DTX, a statistically significant difference (with p < 0.05) was evident beginning at 5 μg/mL, increasing progressively up to 50 μg/mL. This outcome further confirms that BPMO@DTX has superior anticancer effectiveness compared to free DTX and MSN@DTX. Both BPMO and MSN nanoparticles demonstrated negligible or minimal toxicity across the three cell lines: VcAP, A549, and MCF-7.

To evaluate the selectivity of BPMO@DTX and MSN@DTX nanoparticles for cancer cells compared to normal cells, their Selectivity Index (SI) values were calculated based on the IC50 values and are presented in Table S1. Table S1 indicates that across all cancer cell lines tested, BPMO@DTX displayed significantly higher cell selectivity compared to both free DTX and MSN@DTX. Conversely, MSN@DTX did not show any notable improvement in selectivity for cancer cells compared to free DTX. Specifically, in the VcAP cancer cell line, BPMO@DTX exhibited a selectivity index (SI) 2.2 times greater than that of free DTX, demonstrating considerable selectivity. In the A549 and MCF-7 cell lines, the SI value for BPMO@DTX was even higher, approximately 3.70 times that of free DTX. This reinforces the notion that, in comparison to free DTX and MSN@DTX, BPMO@DTX nanoparticles have superior selectivity for cancer cells and represent a promising candidate for cancer treatment (as the SI values for BPMO@DTX are all >10 [22]).

## 4 Conclusions

Here, we successfully synthesized two nanomaterials, MSN and BPMO. Our assessment of their drug loading capability consistently shows that when compared with MSN, BPMO loads DTX more efficiently. In terms of drug release capability, at pH 5.5 and pH 7.4, MSN@DTX's release profile was not significantly improved when compared with free DTX. In contrast, when compared with free DTX, BPMO@DTX exhibited significantly better drug release, indicating its superior performance as a drug carrier. Furthermore, cell toxicity studies revealed that although BPMO@DTX had higher toxicity against VcAP cancer cells when compared with free DTX and MSN@DTX, it did not have significant toxicity and even less toxic against the normal cell line, Hs68. These findings underscore the advantages of the biodegradable BPMO nanomaterial when compared with the non-biodegradable MSN. The great promise showed by BPMO as a nanoparticle carrier for efficient DTX delivery in cancer treatment is attributable to its biodegradability and superior drug release.

## CRediT authorship contribution statement

**Ha Nguyen Van:** Writing – original draft, Project administration, Methodology, Investigation, Formal analysis. **Linh Ho Thuy Nguyen:** Investigation, Formal analysis. **Ngoc Xuan Dat Mai:** Investigation, Formal analysis. **Anh Ha Nhat:** Investigation, Formal analysis. **Trinh Le Thi Thu:** Investigation, Formal analysis. **Anh Nguyen Thi Bao:** Investigation, Formal analysis. **Ha Nguyen Thanh:** Investigation, Formal analysis. **Minh Tri Le:** Supervision, Investigation, Formal analysis. **Tan Le Hoang Doan:** Writing – review & editing, Resources, Methodology, Data curation, Conceptualization.

## Declaration of competing interest

The authors declare that they have no known competing financial interests or personal relationships that could have appeared to influence the work reported in this paper.
