# Supplementary material for: Enhancing docetaxel efficacy and reducing toxicity using biodegradable periodic mesoporous organosilica nanoparticles
Source: Heliyon. 2024 Nov 5;10(22):e40131. doi: 10.1016/j.heliyon.2024.e40131 (PMC11583690; doi:10.1016/j.heliyon.2024.e40131)
Supplement: Multimedia component 1 [file mmc1.docx]

**Supporting Information**

**Enhancing Docetaxel Efficacy and Reducing Toxicity Using Biodegradable Periodic Mesoporous Organosilica Nanoparticles**

Ha Nguyen Van^a,b,d^, Linh Ho Thuy Nguyen^a,d^, Ngoc Xuan Dat Mai^c,d^, Anh Ha Nhat^a,d^, Trinh Le Thi Thu^a,d^, Anh Nguyen Thi Bao^a,d^, Ha Nguyen Thanh^e^, Minh Tri Le^a,b,d^, Tan Le Hoang Doan^c,d,*^

*^a^University of Health Science (UHS), VNU-HCM, Ho Chi Minh City, Viet Nam*

*^b^Faculty of Pharmacy, University of Medicine and Pharmacy at Ho Chi Minh City, Ho Chi Minh City, Viet Nam*

*^c^Center for Innovative Materials and Architectures (INOMAR), Ho Chi Minh City, Viet Nam*

*^d^Vietnam National University, Ho Chi Minh City, Viet Nam*

*^e^Institute of Drug Quality Control Ho Chi Minh City (IDQC HCMC), Viet Nam*

Corresponding author: [dlhtan@inomar.edu.vn](mailto:dlhtan@inomar.edu.vn)


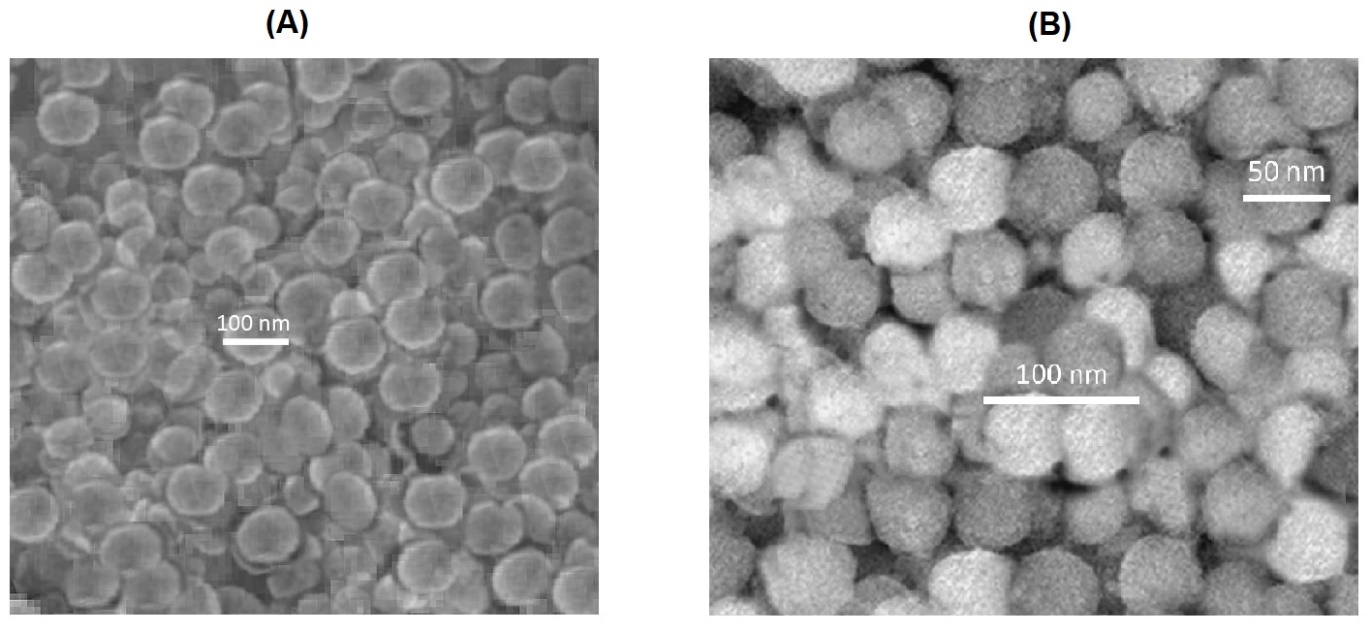


**Figure S1.** SEM images of MSN@DTX (A) and BPMO@DTX (B) nanoparticle

The results indicated that BPMO@DTX (Figure S1A) and MSN@DTX (Figure S1B) maintained their original spherical morphology and particle size, consistent with the initial BPMO and MSN nanoparticles, respectively.

**Table S1.** The IC 50 (µg/mL) and Selectivity Index (SI) of free DTX, BPMO@DTX and MSN@DTX

|  | **Free DTX** | | **BPMO@DTX** | | **MSN@DTX** | |
| --- | --- | --- | --- | --- | --- | --- |
| **Cell lines** | IC 50 (µg/mL) | SI | IC 50 (µg/mL) | SI | IC 50 (µg/mL) | SI |
| **Hs68** | 129.76  ± 2.35 |  | 225.64  ± 4.97 |  | 140.61  ± 3.73 |  |
| **VcAP** | 19.97  ± 0.76 | 6.50 | 15.79  ± 0.83 | 14.29 | 30.65  ± 1.42 | 4.59 |
| **A549** | 17.01  ± 0.51 | 7.63 | 7.99  ± 0.76 | 28.24 | 18.07  ± 1.13 | 7.78 |
| **MCF-7** | 7.93  ± 0.84 | 16.36 | 3.76  ± 0.27 | 60.01 | 8.14  ± 0.46 | 17.27 |

Table S1 indicates that across all cancer cell lines tested, BPMO@DTX displayed significantly higher cell selectivity compared to both free DTX and MSN@DTX. Conversely, MSN@DTX did not show any notable improvement in selectivity for cancer cells compared to free DTX. Specifically, in the VcAP cancer cell line, BPMO@DTX exhibited a selectivity index (SI) 2.2 times greater than that of free DTX, demonstrating considerable selectivity. In the A549 and MCF-7 cell lines, the SI value for BPMO@DTX was even higher, approximately 3.70 times that of free DTX. This reinforces the notion that, in comparison to free DTX and MSN@DTX, BPMO@DTX nanoparticles have superior selectivity for cancer cells and represent a promising candidate for cancer treatment.
